# Bladder Stone Formation on Migrated Hem-o-Lok Clips at the Vesicourethral Anastomosis After Robot-Assisted Radical Prostatectomy: A Case Report

**DOI:** 10.7759/cureus.101512

**Published:** 2026-01-14

**Authors:** Ilias Kanakakis, Athanasios Kopanidis, Louis Giagkou, Ioannis Damakoudis, Vasileios Tzelepis

**Affiliations:** 1 Urology, 417 Army Equity Fund Hospital, Athens, GRC

**Keywords:** bladder calculi, endoscopic removal, hem-o-lok clip, holmium:yag laser, intravesical migration, robot-assisted radical prostatectomy

## Abstract

Robot-assisted radical prostatectomy (RARP) is a well-established surgical treatment for localized prostate cancer, offering excellent oncologic outcomes with reduced perioperative morbidity compared to open surgery. Hem-o-Lok® clips are routinely used during RARP to achieve hemostasis and facilitate precise dissection of the prostatic pedicles. Although generally safe, migration of these non-absorbable clips to the vesicourethral anastomosis represents an uncommon but clinically relevant late complication. Once exposed to urine, migrated clips may serve as a nidus for encrustation and subsequent stone formation, resulting in hematuria or lower urinary tract symptoms (LUTS) that may manifest months or even years after surgery. We present a rare case of bladder stone formation on migrated Hem-o-Lok® clips at the vesicourethral anastomosis four years following RARP, highlighting its clinical presentation, diagnostic workup, endoscopic management, and an alternative mechanical technique for complete clip removal aimed at minimizing the risk of anastomotic injury and recurrence.

## Introduction

Robot-assisted radical prostatectomy (RARP) has become a widely adopted surgical approach for the management of localized prostate cancer, offering excellent oncologic outcomes along with faster recovery, reduced blood loss, and lower perioperative morbidity compared to open surgery. During RARP, non-absorbable polymer Hem-o-Lok® clips are routinely applied for vascular control of the prostatic pedicles and neurovascular bundles. While these clips are highly effective and easy to deploy, their use is not entirely devoid of potential complications.

Among the rare but increasingly recognized postoperative events is intravesical migration of Hem-o-Lok® clips, either to the vesicourethral anastomosis or into the bladder lumen. The mechanism underlying clip migration is multifactorial and may involve incomplete tissue coverage, local ischemia, postoperative urine leakage, and chronic inflammatory changes that can weaken the anastomotic interface [1]. Once exposed to urine, a migrated clip acts as a foreign body, serving as a nidus for calcification and subsequent bladder stone formation [2].

In the single-center series by Zhu et al., clip migration was identified in 26 of 682 patients (3.8%) following RARP, and bladder stone formation occurred in 19 of these 26 cases (73%), indicating that although migration is uncommon, encrustation is a frequent sequela once migration occurs [1].

Clinically, affected patients may present with persistent LUTS, urinary retention, or gross hematuria, symptoms that often mimic more common postoperative conditions, such as bladder neck contracture or recurrent urolithiasis [3]. Consequently, diagnosis may be delayed unless a high index of suspicion is maintained. Awareness of this complication is therefore essential for timely recognition and appropriate management.

In this report, we describe a rare case of bladder stone formation on migrated Hem-o-Lok® clips at the vesicourethral anastomosis following RARP, with particular emphasis on an alternative endoscopic technique that enabled safe and complete clip removal while minimizing the risk of anastomotic injury.

## Case presentation

A 63-year-old male patient with a medical history of type 2 diabetes mellitus and dyslipidemia underwent RARP in 2021 for localized prostate adenocarcinoma (Gleason score 3+4, pT3aN0, cM0). The postoperative course was uneventful, and the patient maintained an undetectable prostate-specific antigen (PSA) level throughout follow-up.

**Figure 1:**
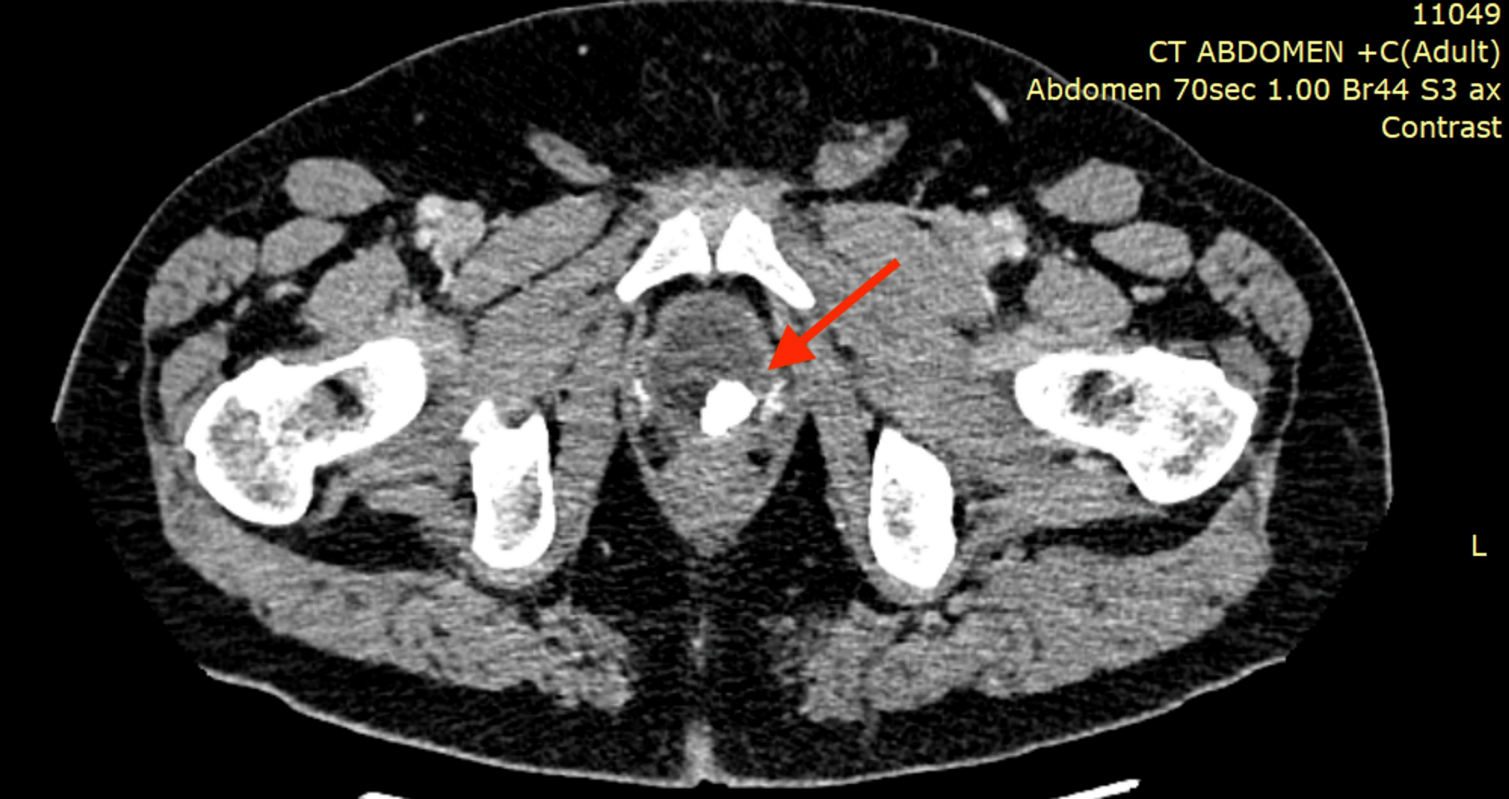
Contrast-enhanced computed tomography (CT) of the pelvis in a transverse plane demonstrating a well-defined hyperdense calcified lesion at the vesicourethral anastomosis (arrow), consistent with a bladder stone formed around migrated Hem-o-Lok® clips.

Four years after surgery, the patient presented with persistent LUTS and intermittent episodes of gross hematuria. Contrast-enhanced computed tomography (CT) of the abdomen and pelvis revealed a 1.7-cm calcified lesion located on the left side of the vesicourethral anastomosis, consistent with a bladder stone (Figure 1). 

**Figure 2:**
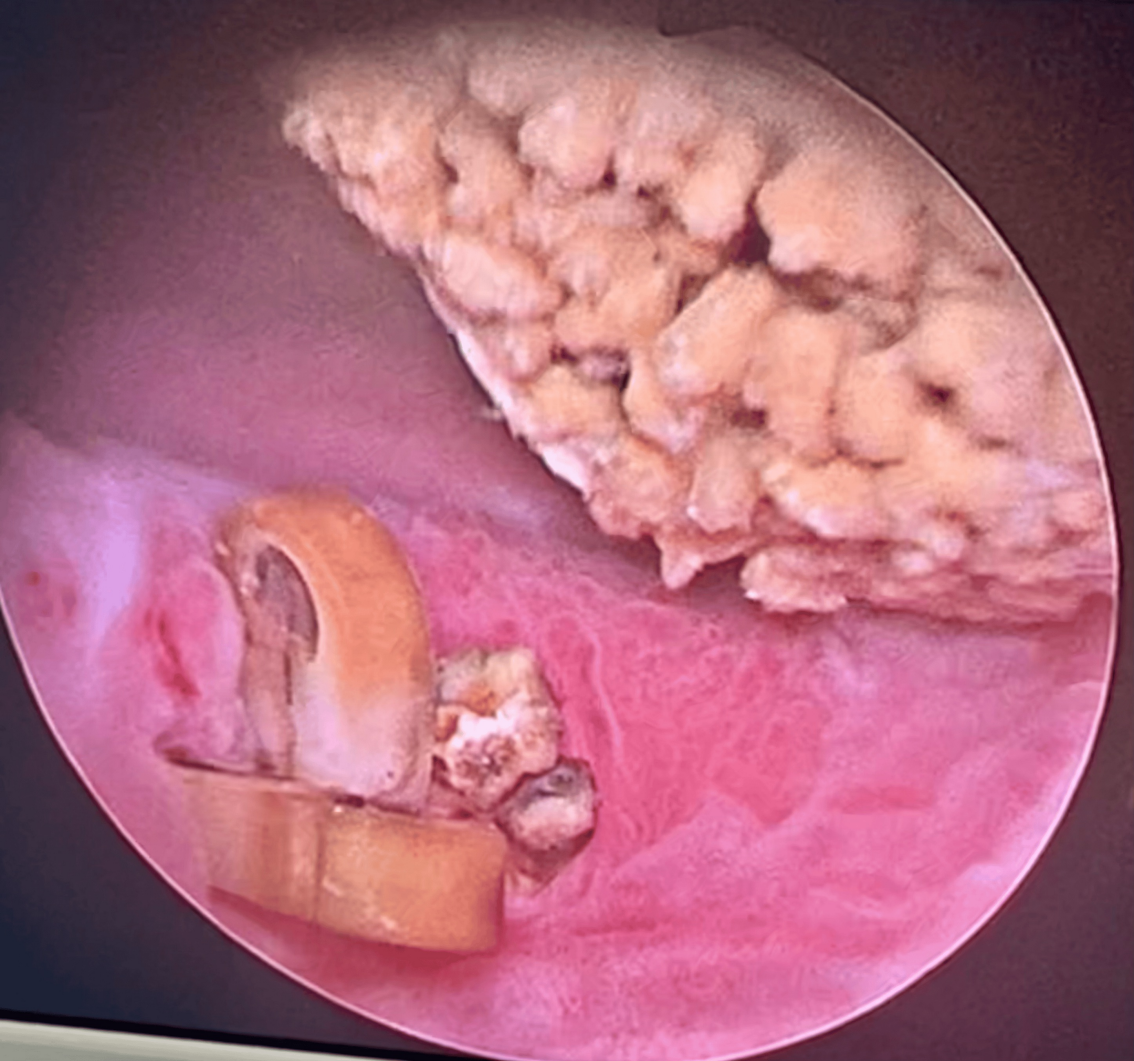
Intraoperative cystoscopic view demonstrating an encrusted Hem-o-Lok® clip embedded at the vesicourethral anastomosis with extensive stone formation, illustrating the clip acting as a nidus for calcification.

The patient subsequently underwent endoscopic cystolithotripsy using a holmium:YAG laser. Intraoperatively, multiple Hem-o-Lok® clips were identified at the vesicourethral anastomosis, acting as the nidus for stone formation (Figure 2). Following laser fragmentation of the calculi, attempts to remove the clips using standard grasping forceps were unsuccessful due to firm adherence to the surrounding fibrotic tissue. 

**Figure 3:**
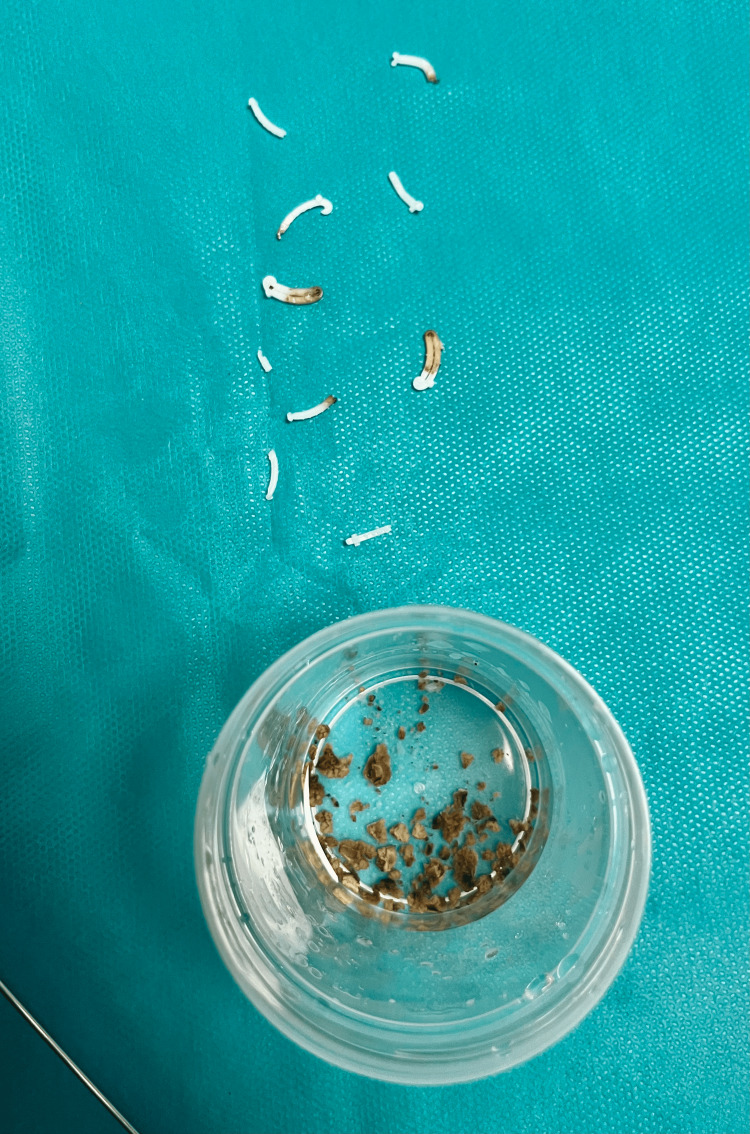
Gross photograph of the extracted Hem-o-Lok® clips and associated bladder stone fragments following endoscopic removal, confirming the clips as the nidus for stone formation.

To avoid additional laser energy delivery or bipolar resection in close proximity to the vesicourethral anastomosis, given the risk of anastomotic injury, postoperative stricture, and urinary incontinence, a purely mechanical approach using a stone punch was employed. This instrument provided superior grip, allowing complete extraction of all embedded clips (Figure 3). A minor bleeding focus was noted at the bed of one clip and was successfully coagulated using the holmium:YAG laser.

The postoperative course was uncomplicated. The urethral catheter was removed on postoperative day one, and the patient was discharged home in good condition without early complications.

## Discussion

Hem-o-Lok® clips are routinely used during RARP for vascular control and hemostasis. While their safety profile is well established, clip migration represents a rare but increasingly recognized late postoperative complication. Clip migration may occur months or even years after surgery and often manifests with nonspecific urinary symptoms, such as LUTS, hematuria, or urinary retention, with a reported mean time to identification of 27.8 ± 18.3 months [4]. Because these clinical features frequently mimic other postoperative conditions, including bladder neck contracture or recurrent urolithiasis, diagnosis may be delayed unless a high index of suspicion is maintained.

Although the overall incidence of clip migration remains low, bladder stone formation is commonly observed following this complication. The chronic presence of a non-absorbable foreign body exposed to urine creates optimal conditions for crystal deposition and progressive encrustation. Cystoscopy remains the gold standard for diagnosis, as imaging studies may not reliably differentiate between a calcified foreign body and a true primary bladder stone [1].

Several mechanisms have been proposed to explain Hem-o-Lok® clip migration. Clip placement in close proximity to the vesicourethral anastomosis, excessive use of electrocautery, postoperative urine leakage, and chronic inflammatory or ischemic tissue changes have all been implicated in promoting erosion and intravesical migration. Progressive fibrosis at the anastomotic site may further destabilize local tissue planes, allowing clips to detach or protrude into the bladder lumen [1].

Recent literature has attempted to categorize Hem-o-Lok® clip migration patterns following prostatectomy. One of the largest reported series described intravesical migration in approximately 0.8% of patients, with a total of 22 documented cases when combined with previously published reports [5]. Based on symptomatology and timing of presentation, migration was classified into three distinct clinical patterns: Type I, characterized by obstructive LUTS occurring within 2-8 months after prostatectomy; Type II, associated with stone formation, gross hematuria, or bladder spasm; and Type III, in which spontaneous clip expulsion was observed within weeks after surgery [5].

From a therapeutic standpoint, endoscopic management remains the treatment of choice. Holmium:YAG laser lithotripsy allows precise fragmentation of encrusted stones with minimal thermal injury. However, when clips are firmly embedded within fibrotic tissue, conventional grasping forceps may be insufficient to achieve safe removal [3].

In the present case, the use of a stone punch provided a purely mechanical alternative, enabling secure grasping and complete extraction of the clips while avoiding excessive laser energy or bipolar resection near the vesicourethral anastomosis. This approach minimized the risk of anastomotic injury, postoperative stricture formation, and urinary incontinence, representing a safe and effective technical modification in selected cases.

Prevention remains a key component in minimizing clip-related morbidity. Recommended strategies include limiting clip use near the bladder neck and prostatic apex, preferring suture ligation for distal pedicles, and ensuring a watertight mucosa-to-mucosa vesicourethral anastomosis to prevent urine leakage and clip exposure [6]. Careful inspection of the anastomotic field at the end of the procedure is essential to confirm the absence of exposed foreign material.

## Conclusions

Hem-o-Lok® clip migration after RARP is an uncommon but clinically relevant complication that may result in secondary bladder stone formation. Awareness of this entity is essential in patients presenting with persistent LUTS or recurrent hematuria long after prostatectomy, allowing timely diagnosis and appropriate management. Meticulous surgical technique remains central to prevention. When stone formation on migrated clips occurs, individualized endoscopic management is required, and our case demonstrates that the use of a stone punch may represent a safe and effective mechanical option for complete clip removal while minimizing the risk of anastomotic injury.
